# Association between maternal hyperglycemia in pregnancy and offspring anthropometry in early childhood: the pandora wave 1 study

**DOI:** 10.1038/s41366-023-01366-6

**Published:** 2023-08-22

**Authors:** Angela Titmuss, Federica Barzi, Elizabeth L. M. Barr, Vanya Webster, Anna Wood, Joanna Kelaart, Marie Kirkwood, Christine Connors, Jacqueline A. Boyle, Elizabeth Moore, Jeremy Oats, H. David McIntyre, Paul Zimmet, Alex D. H. Brown, Jonathan E. Shaw, Maria E. Craig, Louise J. Maple-Brown

**Affiliations:** 1 Wellbeing and Preventable Chronic Diseases Division, Menzies School of Health Research, Charles Darwin University, Darwin, NT Australia; 2 Paediatric Department, Division of Women, Child and Youth, Royal Darwin Hospital, Darwin, NT Australia; 3 Poche Centre for Indigenous Health, University of Queensland, Brisbane, QLD Australia; 4 Clinical and Population Health, Baker Heart and Diabetes Institute, Melbourne, VIC Australia; 5 Endocrinology Department, Division of Medicine, Royal Darwin Hospital, Darwin, NT Australia; 6 Aboriginal Health Domain, Baker Heart and Diabetes Institute, Alice Springs, NT Australia; 7 Northern Territory Department of Health, Darwin, NT Australia; 8 Monash Centre for Health Research and Implementation, School of Public Health and Preventive Medicine, Monash University, Melbourne, VIC Australia; 9 Public Health Unit, Aboriginal Medical Services Alliance of Northern Territory, Darwin, NT Australia; 10 Melbourne School of Population and Global Health, University of Melbourne, Melbourne, VIC Australia; 11 Faculty of Medicine, Mater Medical Research Institute, University of Queensland, Brisbane, QLD Australia; 12 Department of Diabetes, Central Clinical School, Monash University, Melbourne, VIC Australia; 13 University of South Australia, Adelaide, SA Australia; 14 Wardliparingga Aboriginal Research Unit, South Australian Health and Medical Research Institute, Adelaide, SA Australia; 15 Australian National University, Canberra, ACT Australia; 16 Telethon Kids Institute, Perth, WA Australia; 17 School of Women’s and Children’s Health, University of New South Wales, Sydney, NSW Australia

**Keywords:** Obesity, Risk factors

## Abstract

**Background:**

*In-utero* hyperglycemia exposure influences later cardiometabolic risk, although few studies include women with pre-existing type 2 diabetes (T2D) or assess maternal body mass index (BMI) as a potential confounder.

**Objective:**

To explore the association of maternal T2D and gestational diabetes mellitus (GDM) with childhood anthropometry, and the influence of maternal BMI on these associations.

**Methods:**

The PANDORA cohort comprises women (*n* = 1138) and children (*n* = 1163). Women with GDM and T2D were recruited from a hyperglycemia in pregnancy register, and women with normoglycemia from the community. Wave 1 follow-up included 423 children, aged 1.5–5 years (median follow-up age 2.5 years). Multivariable linear regression assessed associations between maternal antenatal variables, including BMI and glycemic status, with offspring anthropometry (weight, height, BMI, skinfold thicknesses, waist, arm and head circumferences).

**Results:**

Greater maternal antenatal BMI was associated with increased anthropometric measures in offspring independent of maternal glycemic status. After adjustment, including for maternal BMI, children exposed to maternal GDM had lower mean weight (−0.54 kg, 95% CI: −0.99, −0.11), BMI (−0.55 kg/m^2^, 95% CI: −0.91, −0.20), head (−0.52 cm, 95% CI: −0.88, −0.16) and mid-upper arm (−0.32 cm, 95% CI: −0.63, −0.01) circumferences, and greater mean suprailiac skinfold (0.78 mm, 95% CI: 0.13, 1.43), compared to children exposed to normoglycemia. Adjustment for maternal BMI strengthened the negative association between GDM and child weight, BMI and circumferences. Children exposed to maternal T2D had smaller mean head circumference (−0.82 cm, 95% CI: −1.33, −0.31) than children exposed to normoglycemia. Maternal T2D was no longer associated with greater child mean skinfolds (*p* = 0.14) or waist circumference (*p* = 0.18) after adjustment for maternal BMI.

**Conclusions:**

Children exposed to GDM had greater suprailiac skinfold thickness than unexposed children, despite having lower mean weight, BMI and mid-upper arm circumference, and both GDM and T2D were associated with smaller mean head circumference. Future research should assess whether childhood anthropometric differences influence lifetime cardiometabolic and neurodevelopmental risk.

## Introduction

Cardiometabolic conditions such as type 2 diabetes (T2D) and obesity are increasing in prevalence worldwide, particularly in youth living in socioeconomically vulnerable settings [1, 2]. Youth-onset T2D and obesity have a higher risk phenotype, pathophysiology and complications than later-onset conditions [3, 4]. The Aboriginal and Torres Strait Islander Australian population, hereafter referred to as First Nations people, have a high prevalence of early-onset cardiometabolic conditions [5, 6]. There is a 10-fold higher prevalence of T2D in pregnancy, as distinct from GDM, among First Nations women than non-Indigenous Australian women [7, 8], and the prevalence of youth-onset T2D is arguably higher than any other population of youth globally in recent years [9].

*In-utero* exposures and early growth are associated with development of later cardiometabolic conditions, such as obesity and T2D [10]. Children exposed to maternal hyperglycemia in pregnancy are at significantly higher risk of later T2D, additive to any risk attributable to adult lifestyle factors [10–15]. There is a continuum of increased risk for adverse cardiometabolic outcomes relating to maternal glucose levels in pregnancy [16, 17]. Altered early growth likely influences the metabolic syndrome pathway, with obesity and T2D both occurring at a younger age [7]. Transgenerational exposure to maternal diabetes and obesity is also associated with cardiometabolic conditions occurring earlier than in preceding generations [18].

Our understanding of the mechanisms by which this increased cardiometabolic risk occurs, however, is incomplete. Most data relate to women with GDM, with only small numbers of women with T2D included in previous studies [19–23]. Children exposed to T2D in pregnancy likely have greater risk of developing youth-onset T2D than children exposed to GDM due to the more severe metabolic changes seen in T2D, as well as early or preconception hyperglycemia and metabolic abnormalities [24]. Preconception abnormalities may occur with both GDM and T2D, however [25], and are associated with epigenetic changes [26]. In addition, few studies have adequately assessed the effect of maternal obesity on the association between maternal hyperglycemia and offspring cardiometabolic risk [19, 20, 27]. Furthermore, few studies exploring maternal hyperglycemia have detailed anthropometric data on young children beyond weight and BMI [28–30]. Other limitations include retrospective study design, reliance on medical record data, heterogeneous classification of hyperglycemia in pregnancy, and short duration of follow-up [27, 30].

The Pregnancy and Neonatal Diabetes Outcomes in Remote Australia (PANDORA) study [8, 31] is a longitudinal cohort of mothers and children living in the Northern Territory (NT) of Australia. The cohort is uniquely positioned to address these evidence gaps as a high proportion of women have T2D and the influence of maternal BMI on the association between maternal hyperglycemia and childhood anthropometry can be assessed prospectively. Data on neonates, previously published by our group, demonstrated increased adiposity in those exposed to T2D in pregnancy [32]. PANDORA also includes a high proportion (59%) of First Nations women and children as participants. Analysis of childhood anthropometry may assist in identifying children at high risk for cardiometabolic conditions, facilitating earlier intervention. This analysis aimed to assess the association of maternal glycemia in pregnancy with anthropometry in early childhood (median age 2.5 years), and whether maternal antenatal BMI is an important confounder of this association.

## Methods

### Participants

**Fig. 1:**
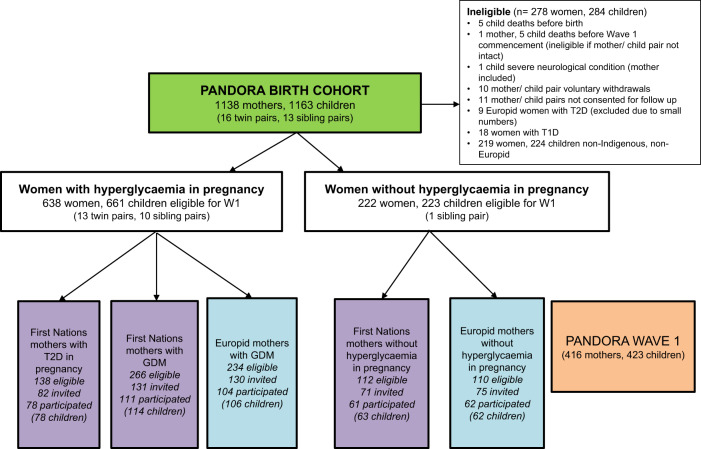
PANDORA Wave 1 study participants.

The PANDORA birth cohort involves 1138 mothers and 1163 children across the NT, Australia, including First Nations, Europid and other ethnicity Australian women (Fig. 1). Women aged ≥16 years with and without hyperglycemia were recruited during pregnancy between November 2011 and February 2017. Women with hyperglycemia (*n* = 903, either T2D, type 1 diabetes or GDM) were recruited from a diabetes in pregnancy register via antenatal clinics. Women with normoglycemia were recruited from antenatal clinics (*n* = 235). Diagnostic criteria for GDM, the recruitment process and eligibility criteria have been described previously [8], with hyperglycemia in pregnancy defined by universal screening during pregnancy. Women with GDM were diagnosed by either 1999 Australian Diabetes in Pregnancy [33] or 2013 World Health Organization (WHO) [34] criteria. The 2013 WHO criteria were used to classify T2D in pregnancy.

A subgroup of cohort women were then invited to participate in the PANDORA Wave 1 follow-up study. Eligible children for Wave 1 (Fig. 1) were aged 1.5–5 years and from five groups, classified by maternal glycemic status and ethnicity. Women with type 1 diabetes (*n* = 18) and Europid women with T2D (*n* = 9) were ineligible for Wave 1 due to small numbers, noting that T2D is uncommon in pregnant Europid women across Australia. Women of other ethnicities (non-Europid and non-Indigenous, *n* = 219) were also ineligible to permit direct comparison between First Nations and Europid populations. Wave 1 was completed in December 2018 and involved 416 mothers and 423 children (255 First Nations and 168 Europid children).

### Maternal and neonatal characteristics (pregnancy and birth assessment)

The following maternal variables were assessed by self-report: ethnicity, location of residence (urban versus remote), smoking in pregnancy (yes/no), alcohol use in pregnancy (yes/no) and educational attainment (completion of 10 years vs <10 years of schooling). Other maternal variables were obtained from medical records: diabetes diagnosis, BMI (from first antenatal visit, adjusted for gestation), gestational weight gain (calculated as the difference between third trimester weight closest to delivery and first measured weight in pregnancy), maternal height at first antenatal visit, parity (0, 1+), age at birth, prevalence of anemia on first antenatal bloods, maternal diabetes treatment modalities and dosage. First Nations women were those who self-identified as Aboriginal and/or Torres Strait Islander.

Neonatal measures included sex, gestational age at delivery, mode of delivery, birth measurements and cord blood c-peptide. Child ethnicity was determined by maternal ethnicity, with 90% of PANDORA neonates born to a First Nations mother also reporting paternal First Nations ethnicity. Details regarding assessment of infant feeding practices are outlined in Supplementary Methods.

### Follow-up child anthropometric assessment (age 1.5–5 years)

Data on child growth were collected directly by study personnel. Anthropometric measures included weight (kg), height (cm), head, mid-upper arm and waist circumferences (cm), and triceps, suprailiac and subscapular skinfold thicknesses (mm) (see Supplementary Methods). Mean interobserver coefficient of variations were comparable to other studies (Table S3) [28].

### Statistical analysis

Statistical analysis was conducted using STATA v15 (Stata Corporation, College Station, TX, USA). Differences in maternal and child characteristics by maternal glycemic status (T2D, GDM, normoglycemia) were assessed. Continuous variables were examined for normal distribution and compared using two sample Student’s *t*-test for normally distributed data and Wilcoxon rank sum test for non-normally distributed data. Pearson’s chi-squared test was used to compare categorical variables. Characteristics of those who participated in Wave 1 were also compared to those who were eligible but did not participate.

Child weight, height, BMI, circumferences and skinfolds were analyzed as continuous measures, adjusted for age and sex in regression models. This was to allow comparisons specific to the study population within the cohort, and acknowledging that growth standards [35] are not available for all anthropometric outcomes.

Multiple linear regression models were used for continuous outcomes, results are reported as regression coefficients (ß estimates) with 95% confidence intervals. Multiple models were considered for all child anthropometric outcomes, in an additive stepwise approach developed to assess whether maternal hyperglycemia and BMI were independent predictors of each child outcome. Covariates included those of the Hyperglycemia and Adverse Pregnancy Outcomes (HAPO) study [21, 36].

Model 1 included maternal glycemia in pregnancy (T2D/ GDM/ normoglycemia), maternal ethnicity, child age, and sex. Birthweight was also adjusted for gestational age at birth.

**Fig. 2:**
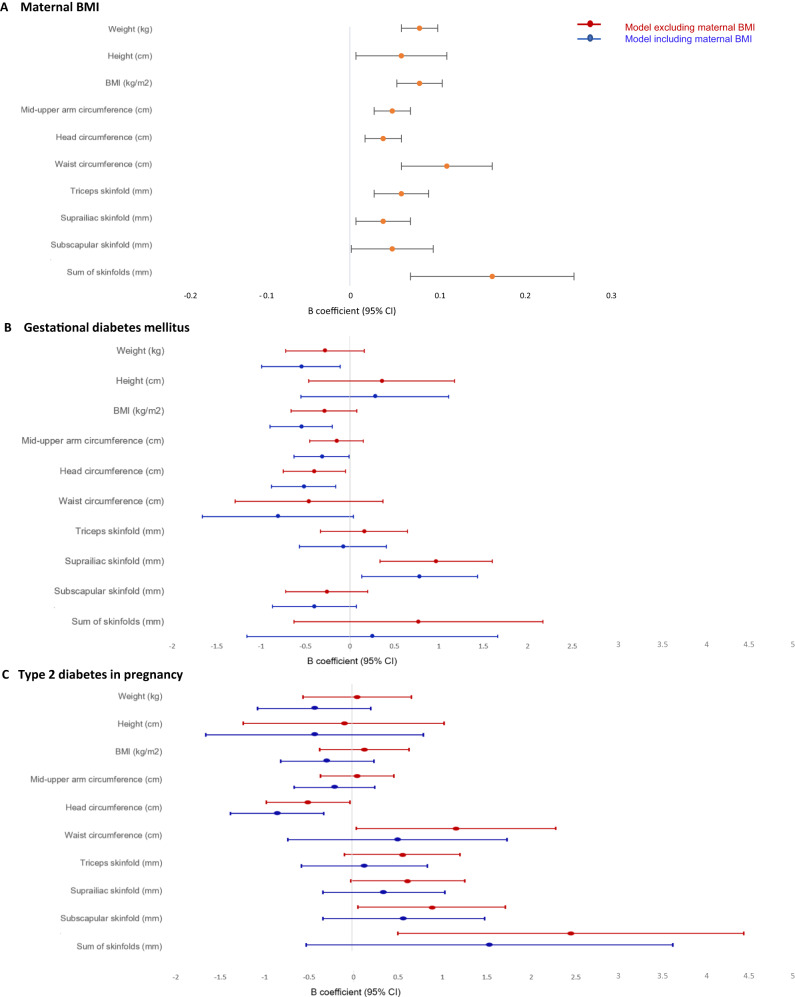
Multivariable analysis of associations of maternal BMI and maternal glycemic status in pregnancy with child anthropometric outcomes. **a** Maternal BMI. **b** Gestational diabetes mellitus. **c** Type 2 diabetes in pregnancy.

Model 2 was as for Model 1, plus adjustment for maternal variables where *p* ≤ 0.2 on univariate analysis. All variables with *p* ≤ 0.2 on univariate analysis (Table S2) were included in the multivariable model building process. Only variables with *p* ≤ 0.1 on stepwise multivariable analysis were included in the final model for each outcome. Maternal ethnicity was included regardless of *p*-value, acknowledging both that Europid women with T2D were excluded and that ethnicity likely represents unmeasured socioeconomic factors. The *p* ≤ 0.1 was chosen to include variables that may have an important confounding effect on other exposures, and to explore variables where, although non-significant, beta coefficient might indicate a significant effect with a larger sample. Therefore, Model 2 for each child anthropometric outcome included different covariates (see footnote Fig. 2).

Model 3 was as for Model 2, plus adjustment for maternal BMI at first antenatal visit, adjusted for gestational age. Additional modeling was also undertaken, again using an additive stepwise approach, to explore the association of maternal BMI with child anthropometry (Table S4).

Analyses were also stratified by ethnicity as the study design only included First Nations women with T2D. Interactions were assessed between maternal glycemic status and ethnicity, and maternal BMI and ethnicity by adding into the models glycemic status by ethnicity, and stratified BMI by ethnicity, as multiplicative terms. A chi-square test for the difference in deviance between the models with and without multiplicative terms was used to assess the statistical significance of the interaction. Collinearity between (i) maternal BMI and height, (ii) maternal BMI and glycemic status and (iii) ethnicity and location of residence, was explored using variance inflation factors (VIF) and correlation coefficients. Sensitivity analyses are detailed in Supplementary Methods.

### Ethics

The study was approved by the Human Research Ethics Committee of the Northern Territory Department of Health and Menzies School of Health Research, and the Central Australian Human Research Ethics Committee. Informed consent was obtained from all women, and from parents/guardians of all children.

## Results

### Demographic characteristics

A total of 423 children had anthropometric measures undertaken and were included in this analysis, at a median age of 2.5 years (range 1.5–5.5 years). The proportion of women with T2D, GDM and normoglycemia differed between those who participated in Wave 1 and those who were eligible but did not participate (Table S1) as preferential sampling was employed to ensure adequate numbers of women from each of the baseline hyperglycemia in pregnancy groups participated. There were no other differences between those who participated in Wave 1 compared to those who were eligible but did not participate.

Among First Nations women, 78 had T2D (31%), 111 GDM (44%) and 61 (24%) normoglycemia in pregnancy (Table 1). Compared to women with GDM, women with T2D had higher cord blood c-peptide results, higher insulin and metformin doses in the third trimester, and were more likely to require a combination of metformin and insulin during the third trimester. Among Europid women, 104 had GDM (63%) and 62 (37%) normoglycemia in pregnancy. Of the 111 First Nations women with GDM, 25 (23%) had glycemic results consistent with the T2D range outside of pregnancy, but diagnosed for the first time in pregnancy [34].

**Table 1:** Characteristics of Pandora Wave 1 women and children by study group.

	First Nations mother					Europid mother			
	T2D (*n* = 78)	GDM (*n* = 111)	Normoglycemia (*n* = 61)	Comparison by maternal glycemic status *p* value	Total (*n* = 250)	GDM (*n* = 104)	Normoglycemia (*n* = 62)	Comparison by maternal glycemic status *p* value	Total (*n* = 166)
Maternal characteristics									
Maternal age at birth (years)	32.0 (5.0)	29.0 (5.6)	24.5 (4.5)	<0.001	28.7 (5.8)	31.8 (5.9)	31.8 (5.4)	0.99	31.8 (5.7)
Gestational age at first weight (weeks)	13.2 (7.2)	14.3 (7.6)	14.0 (7.2)	0.64	13.9 (7.3)	15.1 (5.6)	15.7 (4.4)	0.48	15.3 (5.2)
Maternal BMI at first antenatal visit (kg/m^2^)^a^	31.6 (6.0)	29.1 (7.5)	24.4 (6.4)	<0.001	28.6 (7.3)	29.0 (6.9)	27.2 (5.7)	0.23	28.3 (6.6)
Maternal height at first antenatal visit (cm)	162.6 (5.6)	160.7 (5.4)	163.2 (5.2)	0.01	161.9 (5.5)	164.9 (6.5)	166.2 (5.8)	0.22	165.4 (6.3)
Maternal anemia on first antenatal bloods	9 (12)	12 (11)	16 (25)	0.02	37 (15)	4 (4)	0	0.12	4 (2)
Gestational weight gain (kg)	5.8 (5.0)	7.1 (5.2)	9.7 (5.2)	<0.001	7.4 (5.3)	7.6 (5.9)	10.6 (4.1)	<0.01	8.6 (5.5)
Smoking in pregnancy	30 (39)	42 (40)	24 (40)	0.99	96 (39)	15 (14)	11 (18)	0.57	26 (16)
Remote residence	52 (67)	76 (68)	46 (75)	0.51	174 (70)	3 (3)	0 (0)	0.18	3 (2)
Schooling duration <10 years	23 (30)	21 (20)	6 (10)	0.02	50 (21)	2 (2)	0 (0)	0.27	2 (1)
English as primary language	18 (23)	42 (38)	23 (38)	0.07	83 (33)	103 (99)	61 (98)	0.71	164 (99)
Caesarean delivery	55 (72)	49 (44)	13 (21)	<0.001	117 (47)	37 (36)	19 (31)	0.52	56 (34)
Child characteristics									
Child age at follow-up (years)	3.02 (1.1)	3.0 (1.1)	2.6 (1.0)	0.03	2.9 (1.1)	2.8 (0.7)	2.3 (0.8)	<0.001	2.6 (0.8)
Predominant breastfeeding at six months	24 (59)	59 (81)	41 (79)	0.02	124 (75)	51 (54)	34 (61)	0.40	85 (56)
Child sex (male)	31 (40)	65 (57)	34 (54)	0.05	130 (51)	56 (53)	38 (61)	0.24	94 (56)
Gestational age at birth (weeks)	36.7 (2.5)	38.3 (1.6)	39.6 (1.2)	<0.001	38.1 (2.1)	38.9 (1.4)	39.6 (1.4)	<0.01	39.2 (1.4)
Premature birth (<37 weeks)	26 (33)	15 (13)	1 (2)	<0.001	42 (16)	9 (8)	1 (2)	0.09	10 (6)
Median birthweight (Z score)	0.86 [−0.3, 2.3]	−0.05 [−0.9, 0.8]	−0.18 [−0.9, 0.5]	0.01	0.33 [−0.8, 1.0]	0.18 [−0.5, 0.7]	0.23 [−0.6, 1.0]	0.46	0.19 [−0.5, 0.8]
Cord blood C-peptide (nmol/L)	1.0 (1.1)	0.6 (0.5)	0.4 (0.2)	0.001	–	0.4 (0.2)	0.4 (0.2)	0.28	–
Maternal glycemia characteristics									
Glycated hemoglobin (HbA1c)									
HbA1c (mmol/mol)	60.1 (20.2)	37.3 (5.5)	–	<0.001	–	34.5 (3.0)	–	–	–
Gestational age at HbA1c (weeks)	10.6 (8.7)	22.8 (11.5)	–	<0.001	–	30.1 (8.1)	–	–	–
First oral glucose tolerance test									
Fasting glucose (mmol/L)	–	5.0 (1.1)	4.2 (0.4)	<0.001	–	4.7 (0.7)	4.2 (0.3)	<0.001	–
One hour glucose (mmol/L)	–	9.8 (1.9)	7.1 (1.5)	<0.001	–	9.2 (1.8)	6.8 (1.5)	<0.001	–
Two hour glucose (mmol/L)	–	8.3 (2.0)	6.0 (1.1)	<0.001	–	8.4 (1.5)	5.6 (1.2)	<0.001	–
Gestational age at oral glucose tolerance test (weeks)	–	23.5 (7.1)	26.1 (5.2)	0.01	–	25.4 (5.5)	26.4 (3.8)	0.22	–
Diabetes treatment type				<0.001					
Diet/ lifestyle management only	0	43 (39)	–	–	–	52 (50)	–	–	–
Metformin only	15 (20)	27 (24)	–	–	–	18 (17)	–	–	–
Insulin only	8 (11)	12 (11)	–	–	–	22 (21)	–	–	–
Metformin and insulin	53 (70)	29 (26)	–	–	–	12 (12)	–	–	–
Maximal total daily dose insulin during 3rd trimester (units)	52 [26–79]	18 [10–32]	–	<0.001	–	12 [6–28]	–	–	–
Maximal metformin dose during 3rd trimester (g)	1.8 (0.4)	1.5 (0.6)	–	0.002	–	1.3 (0.8)	–	–	–

Results presented as *n* (%), mean (SD) or median [IQR].

The same measures of severity are not available across categories of maternal hyperglycemia (GDM vs T2D), with oral glucose tolerance test data available for women with GDM and HbA1c data available for women with T2D in pregnancy. Results presented in table refer to that during pregnancy.

Total number of women presented in this table, *n* = 416 (children *n* = 423). Total number is reduced for specific variables:

For First Nations women with T2D, GDM and normoglycemia, respectively: maternal educational attainment, *n* = 76, *n* = 111, *n* = 63; BMI at first antenatal visit, *n* = 69, *n* = 106, *n* = 61; gestational weight gain, *n* = 60, *n* = 97, *n* = 55; smoking in pregnancy, *n* = 75, *n* = 108, *n* = 62; anemia, *n* = 76, *n* = 111, *n* = 63; infant feeding practices, *n* = 41, *n* = 73, *n* = 52; oral glucose tolerance test results, *n* = 104, *n* = 63; cord blood c-peptide, *n* = 41 T2D, *n* = 74 GDM, *n* = 31 normoglycemia. For First Nations women with T2D, HbA1c *n* = 72.

For non-Indigenous women with GDM and normoglycemia respectively: maternal educational attainment, *n* = 104, *n* = 62; BMI at first antenatal visit, *n* = 100, *n* = 60; gestational weight gain, *n* = 79, *n* = 44; smoking in pregnancy, *n* = 104, *n* = 62; anemia, *n* = 104, *n* = 62; infant feeding practices, *n* = 95, *n* = 56; oral glucose tolerance test results, *n* = 102, *n* = 62; cord blood c-peptide, *n* = 63 GDM, *n* = 36 normoglycemia.

*BMI* body mass index, *T2D* type 2 diabetes in pregnancy, *GDM* gestational diabetes mellitus

^a^adjusted for gestational age. Note mean gestational age at time of BMI measurement was 14.4 weeks (13.8, 15.0)

First Nations women were younger than Europid women (Table 1) and more likely to have delivered earlier, smoked during pregnancy, live remotely, and undertaken 10 years or less of secondary schooling (all *p* < 0.001). First Nations and Europid children showed similar sex distribution (*p* = 0.60) but First Nations children were older than Europid children at follow-up (*p* = 0.02). Mean age and sex adjusted weight, height, BMI, and mid upper arm and head circumference were all lower in First Nations than Europid children (all *p* < 0.01) at follow-up, though mean birthweight for gestational age did not differ. Mean sub-scapular and suprailiac skinfold thickness were higher in First Nations than Europid children (*p* < 0.01 and *p* = 0.02, respectively). Mean waist circumference, waist-to-height ratio and triceps skinfold thickness did not vary by ethnicity.

### Association between maternal hyperglycemia in pregnancy and anthropometry of children

Although child BMI did not differ significantly between the maternal glycemic groups among First Nations children on unadjusted comparisons (Table 2), children exposed to T2D *in-utero* had greater mean birthweight Z score (*p* < 0.001), mean waist circumference (*p* = 0.03) and mean skin folds (triceps *p* = 0.04, suprailiac *p* = 0.06, sum of skinfolds *p* = 0.06) compared to children exposed to normoglycemia in pregnancy. First Nations children exposed to GDM had similar anthropometry overall to children exposed to normoglycemia *in-utero*, except for greater mean suprailiac skinfold (*p* = 0.04).

**Table 2:** Anthropometry of Wave 1 children at follow-up by study group, age and sex adjusted.

	First Nations mother						Europid mother			
	All (*n* = 255)	T2D (*n* = 78)	GDM (*n* = 114)	Normoglycemia (*n* = 63)	Comparison by maternal glycemic status (*p* value)		All (*n* = 168)	GDM (*n* = 106)	Normoglycemia (*n* = 62)	Comparison by maternal glycemic status (*p* value)
					T2D vs normoglycemia	GDM vs normoglycemia				GDM vs normoglycemia
Median birthweight Z score [57]	0.33 [−0.8, 1.0]	0.86 [−0.3, 2.3]	−0.05 [−0.9, 0.8]	−0.18 [−0.9, 0.5]	<0.001	0.38	0.19 [−0.5, 0.8]	0.18 [−0.5, 0.7]	0.23 [−0.6, 1.0]	0.46
Child weight (kg)^a^	13.2 (13.0, 14.4)	13.9 (13.3, 14.5)	13.3 (13.0, 13.6)	12.5 (12.1, 13.0)	0.90	0.28	14.6 (14.3, 14.9)	14.7 (14.4, 15.1)	14.0 (13.6, 14.5)	0.17
Child height (cm)^a^	90.4 (90.0, 90.9)	92.1 (91.1, 93.0)	91.4 (90.7, 92.1)	87.9 (87.1, 88.7)	0.40	0.57	93.2 (92.7, 93.8)	94.2 (93.5, 94.9)	90.4 (89.5, 91.2)	0.70
Child BMI (kg/m^2^)^a^	16.1 (15.9, 16.3)	16.2 (15.8, 16.6)	15.9 (15.6, 16.2)	16.1 (15.7, 16.5)	0.40	0.64	16.7 (16.5, 17.0)	16.5 (16.2, 16.8)	17.1 (16.7, 17.5)	0.05
Waist circumference (cm)^a^	48.9 (48.1, 49.8)	51.6 (50.5, 52.6)	49.9 (49.2, 50.5)	49.8 (48.8, 50.8)	0.03	0.94	50.8 (50.2, 51.3)	50.6 (50.0, 51.3)	50.7 (49.8, 51.6)	0.07
Mid upper arm circumference (cm)^a^	16.0 (15.8, 16.2)	16.3 (15.9, 16.6)	16.0 (15.7, 16.2)	15.9 (15.6, 16.2)	0.89	0.32	16.9 (16.7, 17.1)	17.0 (16.7, 17.2)	16.8 (16.4, 17.1)	0.79
Head circumference (cm)^a^	48.4 (48.2, 48.5)	48.3 (48.0, 48.6)	48.5 (48.2, 48.8)	48.1 (47.8, 48.5)	0.37	0.81	50.0 (49.7, 50.2)	49.8 (49.5, 50.1)	50.1 (49.7, 50.5)	0.01
Triceps skinfold (mm)^a^	8.1 (7.8, 8.3)	8.6 (8.0, 9.1)	8.1 (7.6, 8.5)	7.6 (7.0, 8.1)	0.04	0.33	8.6 (8.3, 8.9)	8.6 (8.2, 9.1)	8.4 (7.9, 8.8)	0.74
Subscapular skinfold (mm)^a^	7.1 (6.8, 7.3)	7.6 (7.0, 8.2)	6.8 (6.5, 7.2)	6.9 (6.5, 7.4)	0.14	0.70	6.5 (6.2, 6.8)	6.2 (5.9, 6.6)	6.7 (6.3, 7.1)	0.05
Suprailiac skinfold (mm)^a^	7.1 (6.7, 7.4)	7.4 (6.6, 8.1)	7.4 (6.9, 7.9)	6.2 (5.6, 6.8)	0.06	0.04	6.4 (6.0, 6.8)	6.8 (6.2, 7.3)	5.8 (5.3, 6.3)	0.06
Sum of skinfold thicknesses (mm)^a^	22.05 (21.3, 22.8)	22.98 (21.7, 24.3)	21.91 (20.7, 23.2)	21.01 (19.5, 22.5)	0.06	0.41	21.53 (20.6, 22.5)	21.58 (20.3, 22.8)	21.44 (20.0, 22.8)	0.91

Results presented as mean (95% CI), median [IQR], *n* refers to children.

N.B. 317 (75%) children had skinfold thicknesses measured (First Nations children; *n* = 67 T2D, *n* = 78 GDM, *n* = 46 normoglycemia; Europid children; *n* = 70 GDM, *n* = 56 normoglycemia).

^a^Child age and sex adjusted.

Among Europid children, mean birthweight Z score did not significantly differ across glycemic groups, and children exposed to GDM had lower mean BMI (*p* = 0.05), smaller mean head circumference (*p* = 0.01), smaller mean subscapular skinfold (*p* = 0.05) and greater mean suprailiac skinfold (*p* = 0.06) compared to children exposed to normoglycemia *in-utero*.

Univariate analyses of the associations between potential maternal and child factors and anthropometric outcomes are outlined in Table S2.

### Multivariable analysis

**Fig. 3:**
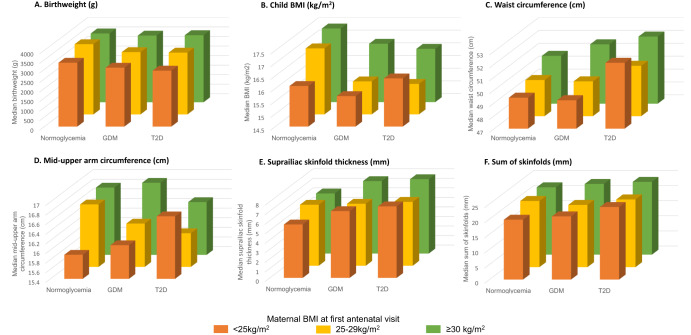
Child anthropometric outcomes by maternal glycemic status in pregnancy and maternal body mass index (BMI) category. **a** Birthweight (g). **b** Child BMI (kg/m^2^). **c** Waist circumference (cm). **d** Mid-upper arm circumference (cm). **e** Suprailiac skinfold (mm). **f** Sum of skinfolds (mm).

Multivariable results (Figs. 2 and 3) have not been stratified by ethnicity as there was no interaction between maternal glycemic status and ethnicity (*p* > 0.05 for all anthropometric outcomes). No collinearity was identified between maternal BMI and height, or maternal BMI and glycemic status (all VIF < 2). Stepwise regression modeling indicated that ethnicity and location of residence were highly correlated with each other (data not shown), thus only ethnicity was included in Models 2 and 3.

After adjustment for maternal variables other than BMI (Model 2), compared to children exposed to normoglycemia *in-utero*, children exposed to maternal T2D had greater mean triceps, suprailiac, subscapular and sum of skinfolds, and greater mean waist circumference. Compared to children exposed to normoglycemia *in-utero*, children exposed to maternal GDM had greater mean suprailiac skinfold thickness. Both GDM and T2D were associated with smaller mean head circumference.

After adjustment for maternal and child factors, including maternal antenatal BMI (Model 3), children exposed to maternal GDM had greater mean suprailiac skinfold thickness (*p* = 0.007), but lower mean weight (*p* = 0.016), BMI (*p* = 0.01), mid-upper arm circumference (*p* = 0.04), and head circumference (*p* = 0.006) than children exposed to normoglycemia *in-utero*. Children exposed to T2D had smaller mean head circumference (*p* = 0.007) and greater mean suprailiac skinfold thickness (*p* = 0.05). Inclusion of maternal BMI in modeling strengthened the association between GDM and mean child weight, BMI and circumferences but attenuated the association with skinfolds. Associations between T2D and child anthropometric outcomes were attenuated after adjusting for maternal BMI. Covariates that remained significant in Models 2 and 3 other than maternal hyperglycemia, BMI and ethnicity included maternal height, parity, smoking in pregnancy and maternal age. Models 2 and 3 for each anthropometric outcome included different covariates (Fig. 2). Multivariable results for all child anthropometric outcomes are presented in Fig. 2.

Maternal BMI was associated with an increase (beta coefficient ranging between 0.03 and 0.17) in all child anthropometric outcomes, independent of maternal glycemic status in pregnancy or ethnicity (Fig. 2). Maternal BMI was most strongly associated with waist circumference (beta coefficient 0.11 cm, 95% CI: 0.06, 0.16) and sum of skinfolds (beta coefficient 0.16 mm, 95% CI: 0.07, 0.25). There was minimal change in the association between maternal BMI and child anthropometric outcomes after adjustment for maternal glycemic status (Table S4). Maternal obesity and hyperglycemia had an additive effect on waist circumference and skinfold thicknesses (Fig. 3). This effect was not seen for child BMI.

For details of sensitivity analyses, see Supplementary Results.

## Discussion

This study describes the anthropometry of children living in the NT, Australia, at a median age of 2.5 years, with 59% being First Nations children, a population at high risk for early-onset cardiometabolic conditions, and with high rates of maternal T2D in pregnancy. The study reports four major findings. Firstly, that greater maternal BMI is associated with increased anthropometric measures in offspring independent of maternal glycemic status. Secondly, that children exposed to GDM had lower mean weight, BMI and upper arm circumferences, and greater mean suprailiac skinfold thickness, compared to children exposed to normoglycemia *in-utero*, after adjustment for maternal factors, including antenatal BMI, and child age and sex. Thirdly, the influence of maternal BMI on associations between maternal hyperglycemia and offspring anthropometry differs between GDM and T2D, strengthening the inverse association between GDM and mean child weight, BMI and circumferences but attenuating the direct associations between T2D and child anthropometry. Fourthly, that both GDM and T2D in pregnancy are associated with smaller mean head circumference in early childhood compared to unexposed children.

Our study indicates that higher maternal BMI is associated with increased anthropometric measures in early childhood, and that BMI partially explains the association between maternal hyperglycemia and some measures, such as sum of skinfolds (Fig. 3). Maternal BMI remained an important predictor in the final multivariable models for all outcomes, independent of maternal hyperglycemia or ethnicity. The association of maternal antenatal BMI with offspring anthropometry, as opposed to maternal hyperglycemia in pregnancy, has been unclear due to limited previous prospective studies [19, 27].

Children born to mothers with GDM had greater mean suprailiac skinfold thickness yet lower mean child weight, BMI, head and mid-upper arm circumferences compared to children of mothers with normoglycemia, even after adjusting for maternal BMI. This is a novel finding, in the context of little consensus regarding the association between maternal hyperglycemia and growth in early childhood [19, 27]. Some studies have indicated children exposed to GDM are heavier and have higher mean BMI than unexposed children [12, 15, 37]. Other data suggest no association between maternal hyperglycemia and childhood anthropometry after inclusion of maternal BMI in modeling [19, 38, 39].

Our study adds to the evidence base with consideration of maternal BMI, more extensive anthropometric measures, and a high risk population compared to studies included in previous meta-analysis [30]. The study suggests possibly reduced postnatal growth after exposure to GDM, leading to lower mean weight, BMI, head and mid-upper arm circumferences in early childhood compared to children exposed to normoglycemia, despite increased measurements at birth [32]. Adjusting for maternal BMI appeared to strengthen the association between GDM and many child anthropometric outcomes, except skinfolds. Greater mean suprailiac skinfold thickness in children exposed to GDM, despite lower mean weight, BMI and circumferences, may reflect altered fat distribution, and have implications for future metabolic risk [28]. This is consistent with the “thin-fat” phenotype described in other populations, where there is preferential growth of adipose tissue compared to fat-free lean mass in children exposed to hyperglycemia in-utero [40–42], compounded by maternal malnutrition [43]. Children exposed to the double burden of malnutrition at different points during pregnancy and their lifecourse, of both nutritional deficiency and dietary excess, are also known to have increased cardiometabolic risk [44]. We note that 50% of First Nations mothers with GDM in our study were treated with metformin, and previous studies have also suggested maternal metformin therapy may influence childhood anthropometry [28].

The study also demonstrated that associations between T2D in pregnancy and some anthropometric measures, such as skinfolds and waist circumference, were attenuated by maternal BMI. This may relate to the differences in BMI between women with T2D compared to those with normoglycemia, as well as the sample size not being calculated for the purpose of comparing differences in anthropometry between T2D and GDM, and so likely underpowered to detect significance. However, few women with T2D have been included in previous studies [15, 19, 45], and the inclusion of 18% of children in our study born to mothers with T2D is a unique strength. Cord blood c-peptide results, as a marker of fuel load on the baby [46], were higher in women with T2D than women with GDM, suggesting more severe insulin resistance and/or hyperglycemia [47]. The differences in treatment modalities, with more women with T2D requiring both metformin and insulin in the third trimester, and at higher doses, than women with GDM, also suggests greater severity of hyperglycemia. Children born to women with T2D may also represent a more heterogenous group in terms of influences on postnatal growth, with T2D associated with both small and large for gestational age at birth. There are likely to be differing associations and causal pathways between maternal BMI and T2D compared to GDM, including differing metabolic changes pre-conception. We acknowledge that some women with GDM may be diagnosed for the first time in pregnancy but, in fact, have had hyperglycemia, obesity and dyslipidemia for many years prior to conception [25]. The degree of hyperglycemia is likely less than in T2D however, contributing to lack of earlier detection. Classification of women by diagnostic categories may therefore reflect severity of exposure for offspring, rather than these exposures being absent prior to diagnosis in women with GDM, as the metabolic changes are a continuum. Further work is required to explore these relationships, and whether maternal BMI is an important determinant of offspring risk, particularly among women with GDM. Children exposed to GDM and normal maternal BMI may have a different risk profile than children exposed to both maternal GDM and obesity [48, 49].

The smaller mean head circumference seen in children exposed to either T2D or GDM, compared to those unexposed, might suggest a possible differential in neurological development [50, 51] requiring further investigation, though the absolute difference was small. Few studies report on head circumference in offspring of women with GDM [28, 39], and none on women with T2D in pregnancy. There is little consensus as to the association between maternal hyperglycemia and neurocognitive risk of children [52], though few women with T2D have been included in previous studies [53]. Head circumference at birth did not vary by hyperglycemia exposure [32], suggesting possibly differential postnatal brain growth between children exposed and unexposed to maternal hyperglycemia.

Our study supports the assessment of anthropometric measures in children beyond BMI. Figure 3 highlights that impacts and relationships of maternal phenotypes vary between different child anthropometric outcomes. We observed an additive effect of maternal obesity and hyperglycemia on several child anthropometric outcomes (birthweight, skinfold thicknesses and waist circumference) however a different pattern was observed for child BMI. These differences may reflect that waist circumference or skinfolds assess different aspects of body composition than child BMI, and thus may have greater utility as measures of cardiometabolic risk.

Of note, First Nations children had greater mean subscapular and suprailiac skinfold thicknesses compared to Europid children, despite lower mean weight, height, BMI and mid-upper arm circumference. This calls into question the utility of BMI in assessing metabolic risk among First Nations Australian children, consistent with previous studies [54]. Lower mid-upper arm circumference, in contrast to greater sub-scapular and suprailiac skinfolds, suggests that fat is more likely to be stored centrally in First Nations children, possibly reflecting increased visceral fat. However, the central fat measures used in this study provide a combined measure of subcutaneous and visceral fat, and thus further exploratory work is required [55]. Previous studies have indicated that childhood skinfold thicknesses and circumferences can serve as proxy indicators of lipid and insulin dysregulation, and that differences in fat deposition, as reflected by BMI, circumferences and fat free mass, can be used to assess cardiometabolic risk [28, 41, 56]. This may indicate higher cardiometabolic risk in First Nations children even from early childhood, consistent with studies from other populations describing the ‘thin-fat’ phenotype [41, 43].

This prospective observational study has explored the impact of maternal hyperglycemia on early childhood anthropometry. Our study is unique, and of relevance to First Nations and transitional populations internationally [57]. A strength of our study is the high proportion of women with T2D in pregnancy, allowing exploration of potentially differential risk between T2D and GDM. Measurement of a variety of anthropometric measures beyond BMI allows more nuanced exploration of cardiometabolic risk, particularly in the context of potentially different fat distribution among First Nations people. The high proportion of First Nations Australians within the cohort, representing a population at high risk of youth-onset cardiometabolic conditions, also allows our study to explore risk factors, including anthropometry and adiposity, from an earlier age.

However, our study has some limitations. Firstly, it was not possible to use dual energy X-ray absorptiometry as a measure of total or percentage body fat [58], because of the remote location and young age of participants. Secondly, the use of a single cross-sectional assessment to assess growth of children is potentially not reflective of their growth trajectory. Thirdly, we acknowledge that hyperglycemia is a continuum, with inherent limitations of analysis by diagnostic categories, though these diagnostic categories are clinically relevant and reflect current practice. We were not able to collect data on glucose levels during pregnancy, though higher cord blood c-peptide results in women with T2D compared to women with GDM or normoglycemia, suggest more severe hyperglycemia and/or insulin resistance in T2D. The impact of treatment of hyperglycemia was also not assessed and may have influenced outcomes. Fourthly, participants may not represent the wider NT population due to the voluntary nature of our study. However, of those on the NT Diabetes in Pregnancy Register, 54% participated in PANDORA, with no significant demographic differences when compared to the clinical register group [8]. Of those eligible and invited, 85% participated in Wave 1, with minimal differences between participants and non-participants (Table S1). Fifthly, it remains difficult to ascertain how maternal BMI may influence the associations between T2D and GDM with child anthropometry as maternal hyperglycemia likely lies on the causal pathway linking maternal BMI to child growth outcomes. Sixthly, paternal data has not been included in analysis but likely impacts on offspring anthropometry. Finally, a longer duration of follow-up is important to determine associations between anthropometry and adiposity in early childhood and later cardiometabolic risk, with risk only becoming apparent over time.

Our study has important implications for clinical practice. The observed differences in childhood anthropometry, with greater mean suprailiac skinfold thickness after exposure to maternal GDM compared to children exposed to normoglycemia, despite lower mean weight, BMI, and mid-upper arm circumferences, are concerning. Exposure to T2D in pregnancy was associated with greater mean skinfold thicknesses across the range of skinfold measures, and greater mean waist circumference, before adjustment for maternal BMI at first antenatal visit. These findings suggest early development of a high-risk “thin-fat” phenotype for later cardiometabolic conditions, with altered adipose distribution, possibly representing increased visceral fat. This requires more investigation, particularly in the context of First Nations Australians already being at much higher risk for cardiometabolic conditions [5, 9, 59, 60]. Maternal hyperglycemia may represent an additional risk factor for these children where early intervention, pre-pregnancy and in early childhood, could decrease cardiometabolic risk [61, 62]. Maternal BMI and hyperglycemia are both modifiable risk factors, and improvements may have beneficial impacts on offspring. Interventions must also address the social and systemic factors contributing to health inequities, and prevent intergenerational transmission of cardiometabolic risk.

In conclusion, maternal BMI was associated with anthropometry of children aged 1.5–5 years independent of maternal glycemia. Children born to mothers with GDM differed in anthropometric measures compared to children exposed to normoglycemia, and inclusion of BMI in modeling strengthened the association with smaller mean weight, BMI, head and mid-upper arm circumferences. Children exposed to T2D in pregnancy had greater mean skinfold thicknesses and waist circumference, and smaller head circumference, with these associations partly explained by maternal BMI. Further work is required to establish whether these changes are correlated with cardiometabolic or neurodevelopmental risk. The study highlights the need to develop effective interventions in childhood to reduce risk, particularly in children born to mothers with hyperglycemia or obesity in pregnancy. It also highlights the role of anthropometric assessment beyond BMI in assessing cardiometabolic risk in children.

### Supplementary information

Supplemental material: Association of maternal hyperglycemia and offspring anthropometry, the PANDORA Wave 1 study

## Data Availability

The datasets generated during and/or analysed during the current study are available from the corresponding author on reasonable request.
