# Supplementary material for: Association between maternal hyperglycemia in pregnancy and offspring anthropometry in early childhood: the pandora wave 1 study
Source: Int J Obes (Lond). 2023 Aug 22;47(11):1120–31. doi: 10.1038/s41366-023-01366-6 (PMC10599996; doi:10.1038/s41366-023-01366-6)
Supplement: Supplementary file 1 — Supplemental material: Association of maternal hyperglycemia and offspring anthropometry, the PANDORA Wave 1 study [file 41366_2023_1366_MOESM1_ESM.pdf]

ASSOCIATION BETWEEN MATERNAL HYPERGLYCEMIA IN PREGNANCY AND OFFSPRING ANTHROPOMETRY IN EARLY CHILDHOOD: THE PANDORA WAVE 1 STUDY

Angela Titmuss[1,2], Federica Barzi[1,3], Elizabeth L.M. Barr[1,4], Vanya Webster[1], Anna Wood[1,5], Joanna Kelaart[6], Marie Kirkwood[1], Christine Connors[7], Jacqueline A Boyle[1,8], Elizabeth Moore[9], Jeremy Oats[10], H. David McIntyre[11], Paul Zimmet[12], Alex D. H. Brown[13,14,15,16], Jonathan E. Shaw[4], Marie E. Craig[17], Louise J. Maple-Brown[1,5].

on behalf of the PANDORA Study research team.

1. *Wellbeing and Preventable Chronic Diseases Division, Menzies School of Health Research, Darwin, NT, Australia*
2. *Paediatric Department, Division of Women, Child and Youth, Royal Darwin Hospital, Darwin, NT, Australia*
3. *Poche Centre for Indigenous Health, University of Queensland, Brisbane, Qld, Australia*
4. *Clinical and Population Health, Baker Heart and Diabetes Institute, Melbourne, Vic, Australia*
5. *Endocrinology Department, Division of Medicine, Royal Darwin Hospital, Darwin, NT, Australia*
6. *Aboriginal Health Domain, Baker Heart and Diabetes Institute, Alice Springs, NT, Australia*
7. *Northern Territory Department of Health, Darwin, NT, Australia*
8. *Monash Centre for Health Research and Implementation, School of Public Health and Preventive Medicine, Monash University, Melbourne, Vic, Australia*
9. *Public Health Unit, Aboriginal Medical Services Alliance of Northern Territory, Darwin, NT, Australia*
10. *Melbourne School of Population and Global Health, University of Melbourne, Melbourne, Vic, Australia*
11. *Faculty of Medicine, Mater Medical Research Institute, University of Queensland, Brisbane, Qld, Australia*
12. *Department of Diabetes, Central Clinical School, Monash University, Melbourne, Vic, Australia*
13. *University of South Australia, Adelaide, SA, Australia*
14. *Wardliparingga Aboriginal Research Unit, South Australian Health and Medical Research Institute, Adelaide, SA, Australia*
15. *Australian National University*
16. *Telethon Kids Institute, Perth, WA, Australia*
17. *School of Women's and Children's Health, University of New South Wales, Sydney, NSW, Australia*

SUPPLEMENTARY MATERIAL

Supplementary Methods:

In this section, we describe our methods in more detail.

The study was approved by the Human Research Ethics Committee of the Northern Territory Department of Health and Menzies School of Health Research, and the Central Australian Human Research Ethics Committee.

Assessment of maternal glycemia

During the course of the study, the GDM diagnostic guidelines were changing both in Australia and internationally. Between 2012-2014, there was a gradual increase in implementation of new guidelines through the Northern Territory (NT). Hence women with GDM were diagnosed by either the 1998 Australian Diabetes in Pregnancy Society (ADIPS)[1] guidelines or a universal 75gm oral glucose tolerance test (OGTT) and revised glucose cut-points as recommended by the International Association of the Diabetes and Pregnancy Study Groups (IADPSG)[2] and the World Health Organization (WHO)[3]. ADIPS 1998 guidelines define GDM when fasting glucose is ≥ 5.5mmol/L or 2-hour level ≥ 8mmol/L.[1] The WHO guidelines define GDM to be when fasting glucose is ≥ 5.1mmol/L or 1-hour glucose ≥ 10.0mmol/L or 2-hour glucose ≥ 8.5mmol/L.[3]

There was an overlap period of approximately two years following the introduction of the new guidelines during which time women were diagnosed with GDM by either of the above guidelines, with formal change over to the new WHO guidelines across the NT in January 2015. Diagnosis of GDM based on these new guidelines included any of the following, a fasting glucose level ≥5.1mmol/L, 1 hour plasma glucose ≥10.0mmol/L or 2 hour plasma glucose ≥8.5mmol/L. Of the PANDORA cohort, 10.3% satisfied only the ADIPS glucose thresholds, 11.5% satisfied only the WHO glucose thresholds and 76.6% satisfied both.

Women were classified as having pre-existing type 1 or type 2 diabetes in pregnancy using WHO criteria,[3] defined as diagnosis using standard diagnostic criteria (OGTT or HbA1c) before the index pregnancy and confirmed on the medical record. Women were classified as having normoglycemia in pregnancy if they did not meet criteria for either GDM or pre-existing diabetes in pregnancy.

An issue when assessing women with GDM is determining women with true GDM as compared to women who have undiagnosed pre-existing type 2 diabetes who were not tested (and so not diagnosed) until the index pregnancy. As per IADPSG[2] and WHO[3] guidelines, women diagnosed with GDM, but meeting glucose or HbA1c values diagnostic of T2D outside of pregnancy were sub-classified as having "diabetes mellitus in pregnancy". This is defined as fasting plasma glucose ≥ 7.0 mmol/L and/or 2-hour plasma glucose ≥ 11.1 mmol/L, or HbA1c ≥ 48mmol/mol (6.5%).

PANDORA Wave 1 participants

A subgroup of women from the PANDORA birth cohort were invited to participate in the PANDORA Wave 1 follow-up study. Eligible children for Wave 1 (Figure 1) were aged 1.5-5 years and from five groups, classified by maternal glycemic status and ethnicity. Women with type 1 diabetes (n=18) and Europid women with T2D (n=9) were not eligible for Wave 1 due to small numbers, noting that T2D is uncommon in pregnant Europid women across Australia. To enable direct comparison between First Nations and Europid populations, women of other ethnicities (non-Europid, non-Indigenous) were also ineligible. There are 884 women with either type 2 diabetes or GDM within PANDORA, of whom 638 were eligible to participate in Wave 1 based on ethnicity and diabetes type, and 235 women in the comparator group, of whom 222 had consented for ongoing follow-up. Only 489 of the 860 eligible women were invited to participate in Wave 1 for the following reasons; i) women residing in extremely remote locations (n=143) or who moved outside of the NT (n=104) were not invited for Wave 1 as it was not logistically feasible at the time to assess them, (ii) peer-reviewed funding was awarded for Wave 1 sample size of n=400, calculated for maternal and child outcomes (iii) children were eligible to be seen from 1.5 years of age and this age was not reached for all children at the time of Wave 1 visits to certain communities. Among women invited, 416 (85%) participated in Wave 1. Preferential

sampling was employed to ensure adequate numbers of women from each of the baseline hyperglycaemia in pregnancy groups participated in Wave 1. Wave 1 was completed in December 2018 and involved 416 mothers and 423 children (255 First Nations and 168 Europid children).

Follow-up child anthropometric assessment (age 1.5-5 years)

Weight was measured using digital scales (Seca Infant Scales, Hamburg, Germany), from children wearing light clothing, no nappies and no shoes. Height was measured as either supine length and standing height in light of the age range of children involved. Supine length was measured from the crown of the head (against a headboard) to a footboard held against the plantar surface of the feet, on a standardized length board (Seca, Hamburg, Germany). Standing height was measured using a standard stadiometer on a level floor (Seca, Hamburg, Germany). Circumferences using a plastic tape measure were taken: 1) Head circumference - above the ears and midway between the eyebrows and hairline to the occipital prominence, 2) Waist circumference at the midpoint of the lowest rib cage and the iliac crest, at the line of the umbilicus in a horizontal plane, in a standing position during end-tidal expiration, 3) Mid upper arm circumference at mid-point between left acromium and the olecranon process. Skinfold thickness was measured on the left side at 3 sites using Holtain calipers (Holtain Ltd, Crosswell, Pembrokeshire, United Kingdom): 1) triceps - midway between the acromion and olecranon, 2) subscapular - lower angle of the scapula, 3) suprailiac - mid-axillary line just above the crest of the ilium. The sum of skinfolds outcome was determined from the triceps, subscapular and suprailiac skinfolds in all children.

Method for each measurement was detailed in the standard operating procedure. Training of study personnel was undertaken by a single person so as to maintain consistency between staff. All measurements were repeated at least twice and the average calculated. A further measurement was made if the difference in measures was ≥0.5cm (height), >0.3cm (circumferences), ≥0.2kg (weight), or >0.5mm (skinfolds), with the average of the two closest measures being used in the analysis.

Infant feeding practices

Breastfeeding data were obtained by either direct telephone or email contact with women at 6–8 months post-partum. The same questionnaire was administered by phone or email. In women for whom direct contact could not be established, data on breastfeeding were obtained through data linkage to primary care electronic medical records at 4–7 months. Predominant breastfeeding was defined as an infant being fed human milk as the only form of milk until 6 months of age consistent with the World Health Organization (WHO) definition, allowing for oral intake of water. It was not possible to determine from the electronic medical record whether breastfeeding was exclusive.

Cord blood c-peptide

Venous cord blood was collected from 245 singleton babies (normoglycemia n = 67, GDM n = 137, T2D n= 41) at delivery. The specimen was obtained by free drainage (free flow) of cord blood or drawn by needle aspiration (puncture) from a clamped segment of an umbilical vein. The specimen was stored in a styrofoam cooler for transport to the hospital's laboratory. Collection of cord blood was not feasible for all participants due to the unpredictable nature of birth and delivery and the need to prioritise clinical care. Cord blood c-peptide was measured using the electrochemiluminescence immunoassay on a cobas e602 (Roche Diagnostics, Mannheim, Germany).

Sample size

Prior to data collection, an assumption was made that Wave 1 would comprise at least 100 women with normoglycemia and 200 with hyperglycemia, and, of the presumed 200 women with hyperglycemia, it was thought that approximately 100 would be Aboriginal women and 100 Europid. At study conclusion in fact there were 123 women with normoglycemia and 293 women with hyperglycemia. Of the 123 women with normoglycemia, 61 women (63 children) were Aboriginal and 62 Europid (62 children). Of the 293 women with hyperglycemia, 189 were Aboriginal women (78 children born to women with T2D and 111 born to women with GDM) and 104 Europid (106 children born to women with GDM). With this number of children, the study had 80% power to detect a minimum difference in child BMI and weight z scores equal to 0.45 SD between the 5 study groups, using a two sample t-test (two-tailed alpha = 0.05).

Statistical analysis

Multiple linear regression models were used for continuous outcomes, results are reported as regression coefficients (ß estimates) with 95% confidence intervals. Multiple models were considered for all child anthropometric outcomes, in an additive stepwise approach developed to assess whether maternal hyperglycemia and BMI were independent predictors of each child outcome.

Model 1 included maternal glycemia in pregnancy (T2D/ GDM/ normoglycemia), child age and sex. Model 2 was as for Model 1, plus adjustment for maternal variables where p value was ≤0.2 on univariate analysis (such as age, smoking in pregnancy (yes/no), parity (0, 1+), alcohol in pregnancy (yes/no), maternal education (≤10 years or > 10 years of schooling), maternal height, gestational age at birth, maternal anemia on first antenatal bloods (yes/no), predominant breastfeeding at six months (yes/no)). All variables with p value ≤0.2 on univariate analysis were included in the multivariable model building process. Only variables with p value ≤0.1 on stepwise multivariable analysis were included in the final model for each outcome. Maternal ethnicity was included regardless of p-value, acknowledging both that Europid women with T2D were excluded and that ethnicity likely represents unmeasured socioeconomic factors. The p value of ≤0.1 was chosen to include variables that may have an important confounding effect on other exposures, and to explore variables that, although non-significant, have a beta coefficient that might indicate a significant effect with a larger sample. Therefore, the final model for each child anthropometric outcome included different covariates (see footnote Table 4). As maternal educational attainment was the socioeconomic measure most consistently associated with outcomes on univariable analyses, it was used in multivariable regression analyses as a marker of socioeconomic status.

Model 3 was as for Model 2, plus inclusion of maternal BMI at first antenatal visit, adjusted for gestational age at the time it was assessed. Analyses were stratified by ethnicity as the study design only included First Nations women with T2D. Interactions were also assessed between maternal glycemic status and ethnicity, and maternal BMI and ethnicity.

Sensitivity analyses

Only 6 Europid women had a First Nations partner. Sensitivity analyses with these women excluded from analysis demonstrated no difference in outcomes, and data are therefore presented by maternal ethnicity.

Women with glycemic results consistent with the T2D range outside of pregnancy, but diagnosed for the first time in pregnancy (n=26, 23%) were included with the GDM group for the main analysis. This is consistent with the current classification guidelines (Australasian Diabetes In Pregnancy Society[4] and World Health Organization[3]), because diagnosis of T2D cannot be definitively confirmed until post-partum. Sensitivity analyses were performed instead including these women in the T2D group. As a number of women did not have weight measured in the third trimester, multivariable models were repeated with the addition of gestational weight gain as a covariate, as well as replacing maternal BMI with gestational weight gain.

In addition, of the 416 women in Wave 1 follow-up, only 317 had adequate breastfeeding data at six months post-partum (108 born to mothers with normoglycemia during pregnancy, 168 born to mothers with GDM, and 41 born to mothers with T2D in pregnancy), reducing sample size. Sensitivity analyses were performed including predominant breastfeeding at 6 months in modelling.

Supplementary Results

Table S1: Comparison of demographic characteristics of women in Wave 1 sub-study to those who did not participate (among those eligible from PANDORA cohort)

	**First Nations mother**			**Europid mother**		
	**Eligible but did not participate** (n=266)	**Wave 1** (n=250)	**p value**	**Eligible but did not participate** (n=178)	**Wave 1** (n=166)	**p value**
**Type 2 diabetes**	62 (23)	78 (31)	0.04	0	0	n/a
**Glycated hemoglobin (HbA1c)** (mmol/mol)	65.2 (23.0)	60.1 (20.2)	0.16	n/a	n/a	n/a
**GDM**	155 (58)	111 (44)	0.002	130 (73)	104 (63)	0.04
**Fasting glucose** (mmol/L)	5.0 (1.1)	5.0 (1.1)	0.99	4.8 (0.9)	4.7 (0.7)	0.30
**One hour glucose** (mmol/L)	9.9 (2.2)	9.8 (1.9)	0.71	9.5 (1.7)	9.2 (1.8)	0.14
**Two hour glucose** (mmol/L)	8.3 (2.2)	8.3 (2.0)	0.76	8.3 (8.0)	8.4 (1.5)	0.66
**Normoglycemia**	49 (18)	62 (24)	0.01	48 (27)	62 (37)	0.04
**Fasting glucose** (mmol/L)	4.2 (0.4)	4.2 (0.4)	0.73	4.2 (0.3)	4.2 (0.3)	0.24
**One hour glucose** (mmol/L)	7.2 (1.6)	7.1 (1.5)	0.74	6.3 (1.3)	6.8 (1.5)	0.07
**Two hour glucose** (mmol/L)	6.0 (1.1)	6.0 (1.1)	0.84	5.5 (1.0)	5.6 (1.2)	0.61
**All**						
**Maternal age at birth** (years)	29.2 (6.3)	28.7 (5.8)	0.36	30.6 (5.2)	31.8 (5.7)	0.05
**Maternal BMI at first antenatal visit**(kg/m^2)	29.4 (6.8)	28.2 (7.1)	0.06	29.3 (7.2)	27.8 (6.4)	0.05
**Gestational weight gain** (kg)	7.6 (6.2)	7.4 (5.2)	0.79	9.2 (5.9)	8.6 (5.5)	0.43
**Smoking in pregnancy**	120 (45)	99 (40)	0.25	28 (16)	26 (16)	0.97
**Remote residence**	181 (68)	178 (71)	0.44	6 (3)	3 (2)	0.36
**Education ≤ 10 years**	53 (20)	49 (20)	0.93	4 (2)	1 (1)	0.46
**Child sex** (male)	142 (51)	130 (51)	0.95	97 (52)	94 (56)	0.51
**Gestational age at birth** (weeks)	38.1 (1.9)	38.3 (2.0)	0.24	39.1 (1.3)	39.3 (1.4)	0.09

Data are mean (SD) or n (%)
Abbreviations: BMI (body mass index); T2D (type 2 diabetes in pregnancy); GDM (gestational diabetes mellitus)

BMI adjusted for gestational age. Note mean gestational age at time of BMI measurement was 14.8 weeks (7.0)

Note: The same measures of severity are not available across categories of maternal hyperglycemia (GDM vs T2D), with oral glucose tolerance test data being available for women with GDM and glycated hemoglobin data being available for women with T2D. Results presented in table refer to that during pregnancy.

Total number of women presented in this table, n=860
Total number is reduced for specific variables: HbA1c, n= 134 (of 138 women with T2D); oral glucose tolerance test results, n= 482 of 500 women with GDM, 221 of 222 women with normoglycemia; BMI at first antenatal visit, n =815; gestational weight gain, n=684; smoking in pregnancy, n= 852.

Of those eligible who did not participate, n= 65 for First Nations women, n=61 for Europid women, who were invited and declined. All others who did not participate were not invited due to study design and feasibility, as outlined in Methods.

Table S2: Univariate analysis of associations between child anthropometric outcomes and potential variables for inclusion in multivariable modelling

Independent variables	CHILD OUTCOMES									
	**Weight (kg)**	**Height (cm)**	**BMI† (kg/m^2)**	**Waist circumference (cm)**	**Mid upper arm circum (cm)**	**Head circum (cm)**	**Triceps skinfold (mm)**	**Suprailiac skinfold (mm)**	**Subscapular skinfold (mm)**	**Sum of skinfolds (mm)**
	B coeff	B coeff	B coeff	B coeff	B coeff	B coeff	B coeff	B coeff	B coeff	B coeff
Maternal hyperglycemia										
None										
GDM	-0.35* (-0.81, 0.12)	-0.04 (-0.92, 0.84)	-0.28* (-0.65, 0.09)	-0.51 (-1.34, 0.33)	-0.14 (-0.46, 0.18)	-0.37* (-1.38, -0.73)	0.12 (-0.38, 0.62)	0.99* (0.36, 1.63)	-0.35* (-0.81, 0.11)	0.51 (-0.87, 1.89)
T2D	-0.67* (-1.27, -0.06)	-1.65* (-2.79. -0.50)	-0.22 (-0.71, 0.26)	0.56 (-0.51, 1.64)	-0.40* (-0.81, 0.02)	-1.18* (-1.67, -0.68)	0.20 (-0.41, 0.81)	1.17* (0.39, 1.95)	0.59* (0.02, 1.16)	1.78* (0.10, 3.47)
Maternal age at birth (years)	0.01 (-0.02, 0.04)	0.06* (-0.01, 0.12)	0.00 (-0.03, 0.02)	0.01 (-0.05, 0.07)	0.00 (-0.02, 0.03)	0.02* (0.00, 0.05)	0.00 (-0.04, 0.03)	-0.03* (-0.08, 0.01)	-0.04* (-0.07, -0.01)	-0.07* (-0.17, 0.03)
Maternal BMI at first antenatal visit (kg/m2)†	-0.02* (-0.06, 0.01)	0.06* (0.00, 0.11)	0.07* (0.05, 0.09)	0.11** (0.06, 0.17)	0.05* (0.03, 0.07)	0.03* (0.01, 0.06)	0.06* (0.03, 0.09)	0.06* (0.02, 0.10)	0.03* (0.01. 0.06)	0.16* (0.07, 0.24)
Gestational weight gain (kg)	0.04* (0.00, 0.09)	0.06* (-0.02, 0.14)	0.00 (-0.03, 0.13)	0.02 (-0.06, 0.10)	0.01* (-0.01, 0.03)	0.03* (0.00, 0.07)	0.01 (-0.03, 0.06)	-0.01 (-0.07, 0.05)	0.01 (-0.04, 0.05)	0.01 (-0.12, 0.14)
First Nations ethnicity	-1.40* (-1.79, -1.01)	-2.80* (-3.54, -2.07)	-0.67* (-0.99, -0.35)	-0.75* (-1.48, -0.02)	-0.93* (-1.19, -0.66)	-1.60* (-1.91, -1.29)	-0.53* (-0.96, -0.10)	0.65* (0.10, 1.21)	0.60* (0.20, 1.01)	0.51 (-0.69, 1.73)
Maternal schooling duration >10 yrs	0.49* (-0.13, 1.110	1.16* (-0.01, 2.32)	0.19 (-0.29, 0.68)	-0.46 (-1.56, 0.63)	0.33* (-0.10, 0.75)	1.01* (0.50, 1.52)	-0.05 (-0.68, 0.58)	-0.61* (-1.45, 0.22)	-0.14 (-0.74, 0.46)	-0.69 (-2.49, 1.11)
Gestational age at birth (weeks)	0.13* (0.03, 0.23)	0.28* (0.09, 0.48)	0.05 (-0.02, 0.09)	0.07 (-0.12, 0.25)	0.10* (0.03, 0.17)	0.13 (0.05, 0.22)	-0.02 (-0.12, 0.09)	-0.06 (-0.19, 0.08)	-0.02 (-0.12, 0.08)	-0.03 (-0.33, 0.27)
Maternal smoking in pregnancy	-0.56* (-0.99, -0.12)	-1.13* (-1.95, -0.30)	-0.17 (-0.52, 0.18)	-0.49 (-1.34, 0.36)	-0.34* (-0.64, 0.05)	-0.80 (-1.16, -0.45)	-0.41* (-0.87, 0.05)	-0.08 (-0.69, 0.52)	0.12 (-0.32, 0.56)	-0.73 (-2.12, 0.67)
Maternal remote residence	-1.33* (-1.71, -0.95)	-2.55* (-3.28, -1.83)	-0.70* (-1.01, -0.39)	-0.61* (-1.32, 0.11)	-0.93* (-1.19, -0.67)	-1.62* (-1.92, -1.32)	-0.46* (-0.88, -0.03)	0.49* (-0.06, 1.03)	0.52* (0.12, 0.92)	0.39 (-0.80, 1.58)
Maternal parity 1+	-0.52* (-0.94, -0.10)	-1.66* (-2.45, -0.86)	0.00 (-0.33, 0.34)	-0.03 (-0.79, 0.73)	-0.21* (-0.50, 0.08)	-0.47* (-0.82, -0.12)	-0.01 (-0.46, 0.44)	0.12 (-0.45, 0.70)	0.11 (-0.31, 0.54)	0.32 (-0.93, 1.56)
Maternal alcohol use in pregnancy	0.17 (-0.30, 0.64)	0.22 (-0.67, 1.12)	-0.02 (-0.03, 0.00)	0.03 (-0.81, 0.87)	0.29* (-0.03, 0.61)	0.61* (0.22, 0.99)	0.54* (0.05, 1.030	0.04 (-0.60, 0.68)	-0.09 (-0.56, 0.37)	0.53 (-0.83, 1.89)
Maternal height (cm)	0.09* (0.06, 0.12)	0.22* (0.16, 0.28)	0.02* (0.00, 0.03)	0.07* (0.02, 0.13)	0.02* (0.01, 0.03)	0.05* (0.03, 0.08)	-0.01 (-0.04, 0.02)	-0.04* (-0.09, 0.00)	-0.01 (-0.05, 0.02)	-0.07* (-0.16, 0.03)
Maternal anemia	-0.78* (-1.46, -0.11)	-1.50* (-2.78, -0.23)	-0.49* (-1.02, 0.04)	-0.73 (-1.93, 0.46)	-0.51* (-0.97, -0.05)	-1.27* (-1.82, -0.71)	-0.69* (-1.42, 0.04)	0.03 (-0.91, 0.98)	0.48* (-0.20, 1.16)	-0.79 (-2.07, 1.91)
Predominant breastfeeding at 6 months	-0.72* (-1.12, -0.31)	-0.90* (-1.80, 0.00)	-0.53* (-0.87, -0.19)	-0.80* (-1.61, 0.00)	-0.50* (-0.80, -0.21)	-0.43* (-0.85, -0.01)	-0.56* (-1.05, -0.06)	0.21 (-0.42, 0.84)	-0.03 (-0.46, 0.41)	-0.26 (-1.57, 1.05)

N.B. All outcomes adjusted for child age and sex, B coeff and 95% CI reported

*p value <0.2

† Maternal BMI adjusted for gestational age at time of measurement

Table S3: Coefficient of variation (CV) in child anthropometric measurements

	**n**	**Mean**	**SD**	**CV (%)**
**Weight** (kg)	423	13.76	3.06	22.2
**Height** (cm)	420	91.55	8.87	9.7
**Waist circumference** (cm)	415	50.35	4.20	8.4
**Mid-upper arm circumference** (cm)	419	16.38	1.54	9.4
**Head circumference** (cm)	419	48.99	2.04	4.2
**Subscapular skinfold thickness** (mm)	295	6.84	1.77	25.8
**Supra-iliac skinfold thickness** (mm)	301	6.79	2.44	35.9
**Triceps skinfold thickness** (mm)	317	8.27	1.96	23.7

Table S4: Associations of maternal BMI at first antenatal visit with child anthropometric outcomes

Outcome	Model 4 [a]			Model 5 [b]			Model 6 [c]		
	B coeff	95% CI	P value	B coeff	95% CI	P value	B coeff	95% CI	P value
**Weight** (kg)	0.08	0.05, 0.10	<0.001	0.08	0.05, 0.1	<0.001	0.08	0.06, 0.11	<0.001
**Height** (cm)	0.06	0.00, 0.11	0.05	0.06	0.01, 0.11	0.019	0.06	0.01, 0.11	0.019
**BMI** (kg/m^2)	0.07	0.04, 0.09	<0.001	0.07	0.05, 0.09	<0.001	0.07	0.05, 0.10	<0.001
**Mid upper arm circumference** (cm)	0.05	0.03, 0.07	<0.001	0.05	0.03, 0.07	<0.001	0.05	0.03, 0.07	<0.001
**Head circumference** (cm)	0.03	0.01, 0.06	0.015	0.03	0.00, 0.05	0.02	0.04	0.02, 0.06	0.001
**Waist circumference** (cm)	0.11	0.06, 0.16	<0.001	0.11	0.06, 0.16	<0.001	0.11	0.06, 0.16	<0.001
**Triceps skinfold** (mm)	0.06	0.03, 0.09	<0.001	0.06	0.03, 0.09	<0.001	0.06	0.03, 0.09	<0.001
**Suprailiac skinfold** (mm)	0.06	0.02, 0.10	0.003	0.06	0.02, 0.1	0.004	0.05	0.01, 0.1	0.018
**Subscapular skinfold** (mm)	0.03	0.01, 0.06	0.020	0.04	0.01, 0.07	0.005	0.04	0.01, 0.07	0.009
**Sum of skinfolds** (mm)	0.15	0.07, 0.24	<0.001	0.17	0.09, 0.26	<0.001	0.16	0.07, 0.25	<0.001

[a] Model 4: Maternal BMI at first antenatal visit (adjusted for gestational age), child age, child sex

[b] Model 5: Model 4 + maternal ethnicity included a priori + maternal variables where p value ≤0.2 on univariate analysis (and then in backward stepwise approach, p value remaining ≤0.1 on regression analysis) Remote residence not included in modelling due to collinearity with ethnicity.

[c] Model 6: Model 5 + Maternal glycemic status in pregnancy (T2D/ GDM/ normoglycemia)

Covariates included for each outcome (in addition to Model 4 + maternal ethnicity + maternal glycemic status):
Weight: maternal height; Height: maternal height, parity; BMI: nil extra; MUAC: nil extra; Head circumference: maternal smoking in pregnancy, anaemia; Waist circumference: maternal height; Triceps skinfold: nil extra; Suprailiac skinfold: nil extra; Subscapular skinfold: maternal age; Sum of skinfolds: maternal age

Sensitivity analyses

Sensitivity analyses including predominant breastfeeding at 6 months in modelling demonstrated no difference in outcomes. Of note, the p-values for predominant breastfeeding at six months for the child outcomes of weight, height, BMI, waist, head and mid-upper arm circumferences, and triceps skinfold were <0.2 on univariate analysis. However, on stepwise multivariable analysis, the p value for predominant breastfeeding at six months was >0.1 for each of the above outcomes and thus not included in the final model of any outcome. To avoid reduced sample size, data are therefore presented without breastfeeding included in modelling.

Women with glycemic results consistent with the T2D range outside of pregnancy, but diagnosed for the first time in pregnancy, were included with the GDM group for the main analysis. Sensitivity analyses, firstly excluding these women, and secondly including these women within the T2D group, demonstrated no differences in outcomes (data not shown). There was no difference in results when gestational weight gain was included in modelling instead of maternal BMI (data not shown).

References

1. Hoffman L, Nolan C, Wilson JD, Oats JJ, Simmons D. Gestational diabetes mellitus--management guidelines. The Australasian Diabetes in Pregnancy Society. Med J Aust. 1998;169(2):93-7.
2. International Association of Diabetes and Pregnancy Study Groups Consensus Panel. International Association of Diabetes and Pregnancy Study Groups Recommendations on the Diagnosis and Classification of Hyperglycemia in Pregnancy. Diabetes Care. 2010;33(3):676-82.
3. World Health Organization. Diagnostic Criteria and Classification of Hyperglycaemia First Detected in Pregnancy. Geneva: World Health Organization; 2013.
4. Rudland VL, Price SAL, Hughes R, et al. ADIPS 2020 guideline for pre-existing diabetes and pregnancy. Aust N Z J Obstet Gynaecol. 2020;60(6):E18-E52.
